# Correction: Laboratory readiness and genomic surveillance of Covid-19 in the capital of Brazil

**DOI:** 10.1371/journal.pgph.0005299

**Published:** 2025-10-08

**Authors:** Fabrício Vieira Cavalcante, Christina Pacheco Santos Martin, Gustavo Saraiva Frio, Rodrigo Guerino Stabeli, Leonor Maria Pacheco Santos

In the Abstract section, there is an error in the second sentence of the paragraph. The correct sentence is: This is a cross-sectional study, with data from: cases/deaths—Ministry of Health; RT-PCR analyses Brasília Central Public Health Laboratory (LACEN); genomics—Global Initiative on Sharing All Influenza Data (GISAID).

In the Method section, there is an error in the first sentence of the first paragraph. The correct sentence is: This is a cross-sectional, exploratory study, based on secondary data in the public domain.

In the Discussion section, there is an error in the second sentence of the seventh paragraph. The correct sentence is: This lag time is within not the time parameters for releasing results within three days, as explained by the Ministry of Health [36].

## References

[1] Cavalcante FV, Martin CPS, Frio GS, Stabeli RG, Santos LMP. Laboratory readiness and genomic surveillance of Covid-19 in the capital of Brazil. PLOS Glob Public Health. 2025;5(1):e0003289. doi: 10.1371/journal.pgph.0003289 39787152 PMC11717220

